# Tau- but not Aß -pathology enhances NMDAR-dependent depotentiation in AD-mouse models

**DOI:** 10.1186/s40478-019-0813-4

**Published:** 2019-12-09

**Authors:** Enrico Faldini, Tariq Ahmed, Luc Bueé, David Blum, Detlef Balschun

**Affiliations:** 1 Brain & Cognition, Faculty of Psychology and Educational Sciences, K.U.Leuven, Tiensestraat 102, 3000 Leuven, Belgium; 2 Qatar Biomedical Research Institute, Hamad Bin Khalifa University, Doha, 34110 Qatar; 3 Jean-Pierre Aubert research centre UMR-S1172, Université de Lille, Inserm, CHU-Lille, LabEx DISTALZ, Alzheimer & Tauopathies, 59045 Lille, France

**Keywords:** Alzheimer’s disease, Synaptic plasticity, Depotentiation, Hippocampus, CA1-region, Tau, Aβ, Glycogen synthase kinase-3β

## Abstract

Many mouse models of Alzheimer’s disease (AD) exhibit impairments in hippocampal long-term-potentiation (LTP), seemingly corroborating the strong correlation between synaptic loss and cognitive decline reported in human studies. In other AD mouse models LTP is unaffected, but other defects in synaptic plasticity may still be present. We recently reported that THY-Tau22 transgenic mice, that overexpress human Tau protein carrying P301S and G272 V mutations and show normal LTP upon high-frequency-stimulation (HFS), develop severe changes in NMDAR mediated long-term-depression (LTD), the physiological counterpart of LTP. In the present study, we focused on putative effects of AD-related pathologies on depotentiation (DP), another form of synaptic plasticity. Using a novel protocol to induce DP in the CA1-region, we found in 11–15 months old male THY-Tau22 and APPPS1–21 transgenic mice that DP was not deteriorated by Aß pathology while significantly compromised by Tau pathology. Our findings advocate DP as a complementary form of synaptic plasticity that may help in elucidating synaptic pathomechanisms associated with different types of dementia.

## Introduction

Alzheimer’s disease (AD) is a multifactorial neurodegenerative syndrome causing most cases of dementia. The understanding of AD has greatly benefitted from the study of animal models expressing human transgenes that contain disease-linked mutations in amyloid precursor protein (APP), presenilins (PSE) 1 and 2, or Tau [4, 5, 63]. These mutations drive the development of AD pathological hallmarks such as the production of toxic Aβ-species, extraneuronal deposition of amyloid plaques as well as hyperphosphorylation of Tau protein and intraneuronal accumulation of neurofibrillary tangles (NFTs), recapitulating critical traits of AD in humans [3, 62, 64, 65].

Memory loss, one of the earliest cognitive symptoms of AD, is closely correlated with synaptic pathology [50]. Given that use-dependent modification of synaptic efficacy (synaptic plasticity) is probably the most ubiquitous mechanism for encoding memories at the cellular level [45], the assessment of synaptic plasticity constitutes a crucial step in determining how abnormalities at synapses cause memory deficits in animal models. This has been mostly verified by measuring long-term potentiation (LTP) in the hippocampal CA1 area, demonstrated to be required for behavioral learning and memory [14, 51, 77]. Several AD transgenic rodent models present compelling proof of the association between deficient LTP at, or even before, the onset of histopathological AD symptoms and memory loss (see [43, 66, 68, 69, 71] and references therein). Abnormal use-dependent *weakening* of synaptic transmission, namely ‘long-term-depression’ (LTD), has also been reported. For instance, Aβ peptides enhance hippocampal LTD *in vitro* [11, 31, 37]. In transgenic APP mice, early accumulation of Aβ facilitates LTD [8, 13] while Aβ prevents its induction at later stages [9, 68]. Recently, we have documented that NMDAR-dependent LTD is abolished in the THY-Tau22 mouse model of tauopathy [2, 35, 73].

While a wealth of studies reported on changes of LTP and LTD in AD mouse models, few, if any, focused on ‘depotentiation’ (DP), the activity-induced reversal of LTP. DP and LTD are the necessary counterparts of LTP [45], and in the hippocampal CA1-region, DP requires the integrity of NMDAR and/or metabotropic glutamate receptors, and of intracellular second messenger systems known to be pathologically modified by Aβ or Tau pathologies (see [61] for a review). Furthermore, DP is naturally observed *in vivo* [78], may occur as ubiquitously as LTP [74], and has been implicated in cellular memory erasure [1, 30, 47].

Surprisingly, to the best of our knowledge, only one study has previously evaluated DP in an AD mouse model. Huh et al. [26] presented mixed results using Tg2576 mice which express the APPswe mutation. In this study, DP could not be induced in 14–19 month-old mice, but was normal when mice were 6–7 months-old. The aim of the present study was, therefore, twofold. Firstly, to test DP in APPPS1–21, an advanced amyloidosis mouse model that displays an earlier onset of amyloid deposition, as well as a higher Aβ42–40 ratio compared to single-mutant APP transgenic mice such as Tg2576 [58]. Secondly, to evaluate how DP is affected by Tau pathology, as presented by THY-Tau22 transgenic mice, an established tauopathy model relevant for AD research [36, 63, 73]. To this end, we further characterized our recently established DP-induction protocol that employs physiological patterns of electrical stimulation [34] and assessed DP in the hippocampal CA1-region at a comparable age as used in earlier studies to examine LTP and LTD [2, 17, 63].

## Materials and methods

### Wild-type mice

In experiments involving only wild-type mice, 2–3 month-old, 6–9 month-old or 17–19 month-old C57BL/6 J of both genders were used (Elevage Janvier, Le-Genest-Saint-Isle, France). Mice were group housed in standard animal cages (12 h/12 h light-dark cycle, 22 °C, ad libitum food and water access), and were allowed to adapt to their new environment after transportation for at least two weeks before experimentation.

### APPPS1–21 transgenic mice

APPPS1–21 heterozygous male mice (APPPS1–21 TG) and C57BL/6 J male littermates (APPPS1–21 WT) were provided by Bart De Strooper (Laboratory for the Research of Neurodegenerative Diseases, University of Leuven, Belgium). As previously described [58], the strain was generated by co-injecting APPKM670/671NL and PS1L166P constructs into male pro-nuclei of WT oocytes. APPPS1–21 TG co-express human amyloid precursor protein ‘Swedish’ (APP_swe_) and presenilin (PS1) mutations under control of a Thy1 promoter that restricts expression to postnatal brain, achieving high levels of neuron-specific transgene expression [58]. APPPS1–21 TG were backcrossed to C57BL/6 J for 8–12 generations. Offspring was genotyped using PCR on DNA isolated from tail biopsy. Mice were aged 13–15 month-old in experiments here described, an age in which amyloid pathology is consolidated to the point of resulting in clear synaptic plasticity abnormalities [17] (also NMDAR-LTD, unpublished data). In addition, in our hands, slightly younger APPPS1–21 TG mice (9–10 months) do not necessarily show an advanced phenotype in regards to learning and memory deficits [40, 41].

### THY-Tau22 transgenic mice

THY-Tau22 heterozygous male mice (THY-Tau22 TG) and C57BL/6 J male littermates (THY-Tau22 WT) were provided by David Blum and Luc Buée (INSERM UMR-S1172, Lille, France). The Tau mutations G272 V and P301S were generated by site-directed mutagenesis PCR into the human 4-repeat Tau cDNA as previously described [63]. This model overexpresses mutated human Tau under the control of a Thy 1.2 promoter that specifically drives expression in neurons starting at postnatal day 6 and thus not directly affecting embryonic development. The vector was injected into a C57BL6/CBA background and backcrossed to C57BL/6 J for > 30 generations. Offspring was genotyped using PCR on DNA isolated from tail biopsy [63]. Mice were aged 11–13 month-old in experiments here described, the same age in which the full AD-like spectrum of tau pathology was noticeable, and in-between younger (6–7 months) and older (14–15 months) ages when HFS-LTP was still normal [63].

### Slice preparation

Mice were killed by cervical dislocation and hippocampus (HC) was rapidly dissected out into ice-cold (4 °C) artificial cerebrospinal fluid (ACSF), saturated with carbogen (95% O_2_ / 5% CO_2_). ACSF consisted of (in mM): 124 NaCl, 4.9 KCl, 24.6 NaHCO_3_, 1.20 KH_2_PO_4_, 2.0 CaCl_2_, 2.0 MgSO_4_, 10.0 glucose, pH 7.4. Transverse hippocampal slices (400 μm thick) were prepared from the dorsal area of the right HC with a tissue chopper and placed into a submerged-type chamber, where they were kept at 32 °C and continuously perfused with ACSF at a flow-rate of 2.4 ml/ min.

### Electrophysiology

After 90 min incubation, a bipolar tungsten electrode was placed in CA1 *stratum radiatum* for stimulation and a glass electrode (filled with ACSF, 3–7 MΩ) about 200 μm apart for recording of field excitatory postsynaptic potentials (fEPSPs). Signals were amplified by a differential AC Amplifier Model 1700 (A-M Systems), fed through a Power1401 data acquisition interface (Cambridge Electronic Design Limited) and analyzed by custom-made software. The time course of the fEPSP was calculated as the descending slope function for all experiments. After input /output curves (I/O) had been established, the stimulation strength was adjusted to elicit a fEPSP-slope of 35% of the maximum and kept constant throughout the experiment. During baseline recording, three single responses were evoked at a 10 s interval by biphasic stimulation (0.1 ms pulse width) and averaged. These measurements were repeated every 5 min.

### LTP induction protocol

To induce an unsaturated form of LTP, a single **T**heta-**B**urst-**S**timulation (TBS) was employed, consisting of 10 bursts of four stimuli at 100 Hz separated by 200 ms (double pulse width) followed by recording of evoked responses at 1, 4, 7 and 10 min post TBS delivery. Thereafter, recording was continued every 5 min until the end of experiments. In case DP induction followed the induction of LTP, evoked responses at 1, 3 and 5 min were measured after TBS, before application of TPS.

### DP induction protocol

6 min after the induction of LTP, DP induction was attempted by delivery of increasingly larger TPS trains (**T**heta-**P**ulse-**S**timulation; continuous stimulation with single pulses at 5 Hz; 2 min-DP2; 3 min-DP3; 5 min-DP5; 8 min-DP8). As a result of these parametric studies (see ‘Results 3.1 and 3.2’), TPS of 8 min duration (DP8) was set as standard for the remaining series of experiments. After DP induction, evoked responses were recorded 1, 4, 7 and 10 min and thereafter every 5 min until the end of experiments.

### Drug application

Drugs were obtained from Abcam PLC (Cambridge, UK) and stored as stock solutions at -20 °C until the day of the experiment, when they were dissolved to the desired final concentration in ACSF and applied via the perfusion line. An involvement of N-Methyl D-Aspartate receptors (NMDAR) in DP induction was tested with the widely used competitive NMDA receptor antagonist (2*R*)-amino-5-phosphonovaleric acid (D-AP5; 50 μM). To ensure that the drug did not interfere with LTP induction, D-AP5 was applied from 6 min prior to TPS (i.e., immediately after TBS) until 15 min after TPS application. A requirement for glycogen synthase kinase-3β (GSK3β) activity in DP induction in wild-type mice, or as a mediator of putative DP phenotypes of APPPS1–21 and/or THY-Tau22 TG [2, 27, 67], was tested with the selective GSK3 inhibitor 3-(2,4-Dichlorophenyl)-4-(1-methyl-1H-indol-3-yl)-1H-pyrrole-2,5-dione (SB216763; 10 μM). SB216763 was added 30 min prior to LTP induction until 15 min after TPS application. Drug experiments were interleaved between genotypes, and drugs and vehicle controls.

### Statistics

All data are presented as mean ± standard error of mean (SEM), where “n” refers to the number of animals tested. Differences between mean values (time series) were examined with two-way analysis of variance with repeated measures (RM-ANOVA) and Holm-Sidak procedures for post hoc comparisons. All other group comparisons were calculated with two-tailed unpaired Student *t*-test with Welch correction or one-way ANOVA with Fisher’s LSD method for pairwise comparisons. Curves of the rise kinetics of fEPSP potentiation were obtained by nonlinear regression with the eq. *Y = Y*_*0*_ + a*(1-exp (−τ*x) (one-phase exponential rise with the asymptote *y*_o_, the span *a* and the rise-time constant τ), using GraphPad Prism 4.0 software (GraphPad Software, Inc., San Diego, CA). Data for curve fitting comprised recordings obtained post-TPS application, yielding τ_rise_ and ‘plateau levels’ of each single DP experiment. Differences with *p* ≤ 0.05 were considered statistically significant.

## Results

### The magnitude of DP in 2–3 month-old mice depends critically on the duration of TPS

**Fig. 1:**
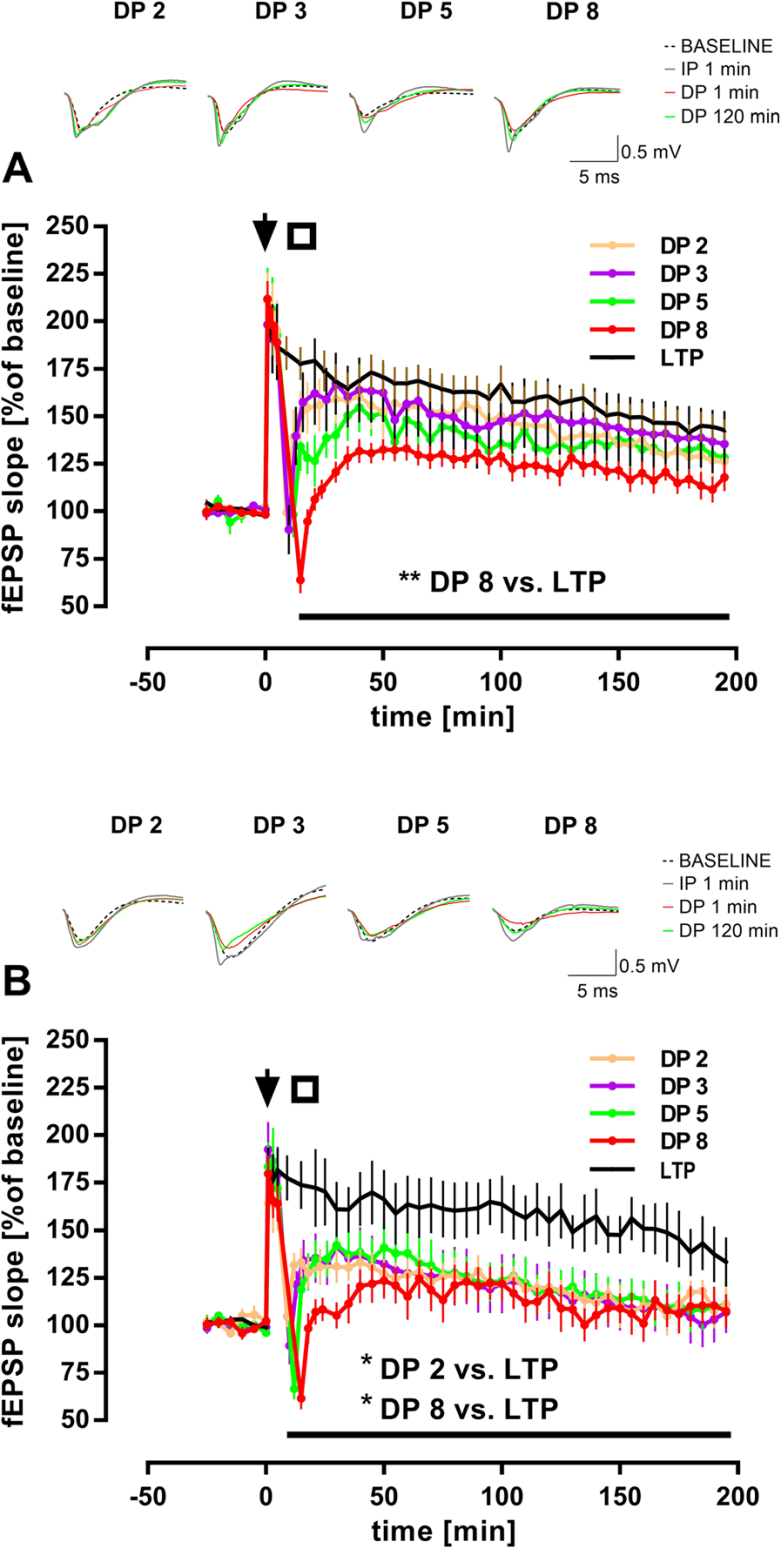
Hippocampal depotentiation in adult wild-type mice induced by theta-patterned stimuli. (**a**) After application of a single theta-burst-stimulation (TBS; indicated by arrow) to induce long-term-potentiation (LTP) in CA1 of 2–3 month-old C57Bl/6 J mice, the induction of depotentiation (DP) was attempted by delivery of theta-pulse-stimulation episodes (TPS; indicated by an open square) of either 2 min (DP 2; *n* = 9), 3 min (DP 3; n = 9), 5 min (DP 5; *n* = 7) or 8 min (DP 8; n = 7). While a ‘dose-response’ relationship could be observed, wherein longer TPS trains evoked increasingly stronger reversal of LTP, only DP 8 induced robust DP when compared to LTP controls (*n* = 10) (*F*_(1, 15)_ = 15.14, **p = 0.001). Error bars represent SEM. Traces show representative examples of field excitatory postsynaptic potentials (fEPSP) recorded during baseline, 1 min post-TBS delivery (IP 1 min), 1 min post-TPS delivery (DP 1 min), and 120 min post-TPS delivery (DP 120 min). Calibration bars: 0.5 mV and 5 ms. (**b**) Replicating same experiments with 6–9 month-old C57Bl/6 J mice revealed a susceptibility of DP to ageing. DP was not dependent on duration of TPS, since both DP 2 (*n* = 6) and DP 8 (n = 6) significantly reversed LTP (DP 2: *F*_(1, 12)_ = 4.867, *p = 0.047; DP 8: *F*_(1, 12)_ = 7.037, *p = 0.021), while DP 3 (n = 6) and DP 5 (n = 6) also showed a trend to significant DP when compared to LTP controls (*n* = 8) (DP 3: *p* = 0.073; DP 5: *p* = 0.074). Error bars represent SEM. Traces show representative examples of field excitatory postsynaptic potentials (fEPSP) recorded during baseline, 1 min post-TBS delivery (IP 1 min), 1 min post-TPS delivery (DP 1 min), and 120 min post-TPS delivery (DP 120 min). Calibration bars: 0.5 mV and 5 ms

Early studies reported that DP could be induced by TPS following the induction of TBS-LTP in the hippocampal CA1 region of adult rats [33, 70]. We first examined whether it was possible to establish a robust protocol to induce DP in adult mice (2–3 months old) using the same stimulation paradigms. TPS episodes of increasing duration revealed a ‘dose-response’ relationship where the effectiveness of inducing DP was proportional to the duration of TPS (Fig. 1a). DP2 (*n* = 9), DP3 (n = 9) and DP5 (*n* = 7) depressed fEPSPs transiently after conditioning as indicated by significant *conditioning* by *time* effects (_**DP 2** × LTP_: F (38, 646) = 5.501, *p* < 0.0001; _(**DP 3** × LTP)_: F (38, 646) = 7.559, p < 0.0001; _(**DP 5** × LTP)_: F (38, 570) = 3.605, p < 0.0001). However, fEPSP potentiation in all above conditions returned to levels that were statistically indistinguishable from LTP controls (*n* = 10), despite that in DP5 a trend to a significant conditioning effect could be observed (main effect of *conditioning*_(**DP 2** × LTP)_: *p* = 0.245; _(**DP 3** × LTP)_: *p* = 0.513; _(**DP 5** × LTP)_: *p* = 0.063). In contrast, significant levels of DP were achieved with DP8 (*n* = 7) (main effect of *conditioning*_(**DP8** × LTP)_: F _(1, 15)_ = 15.14, *p* = 0.001). From these experiments, it is clear that TPS can reverse TBS-LTP in adult mice insofar as long stimulation trains are employed. Under our experimental conditions, DP8 causes significant reversal of fEPSP potentiation.

### The modification range of DP is reduced in 6–8 month-old mice

While DP in CA1 was reported initially as an age-independent form of synaptic plasticity [48, 53, 75], no parametric studies assaying TBS and TPS together addressed this issue before. Thus, in a second series of experiments, we tested DP induction in slices from slightly older mice (6–9 months) employing the same parameters as above. In contrast to the results obtained with 2–3 month-old mice, in 6–9 month-old mice already the shorter TPS trains (DP2, DP3, DP5) yielded DP of similar strength, that was either siduced robust LTP reversal when compared to LTP congnificantly different from LTP controls (*n* = 8) (Fig. 1b; DP2, *n* = 6: main effect of *conditioning*_(**DP2** × LTP)_: F _(1, 12)_ = 4.867, *p* = 0.047), or approached significance, due to a slightly higher biological variability within the particular group (DP3, *n* = 6: main effect of *conditioning*_(**DP3** × LTP)_: F _(1, 12)_ = 3.862, *p* = 0.073; DP5, n = 6: main effect of *conditioning*_(**DP5** × LTP)_: F _(1, 12)_ = 3.828, *p* = 0.074). As in the younger animals, statistically significant reversal of LTP was clearly achieved with DP8 (n = 6) (main effect of *conditioning*_(**DP8** × LTP)_: F _(1, 12)_ = 7.037, *p* = 0.021). The above data point to a facilitated LTP reversal when TPS is delivered shortly after TBS in 6–9 months mice. Based on these and the results with 2–3 months-old mice, we determined our standard protocol for the remaining studies to be DP8.

### Inhibition of GSK3β does not interfere with NMDAR-DP

**Fig. 2:**
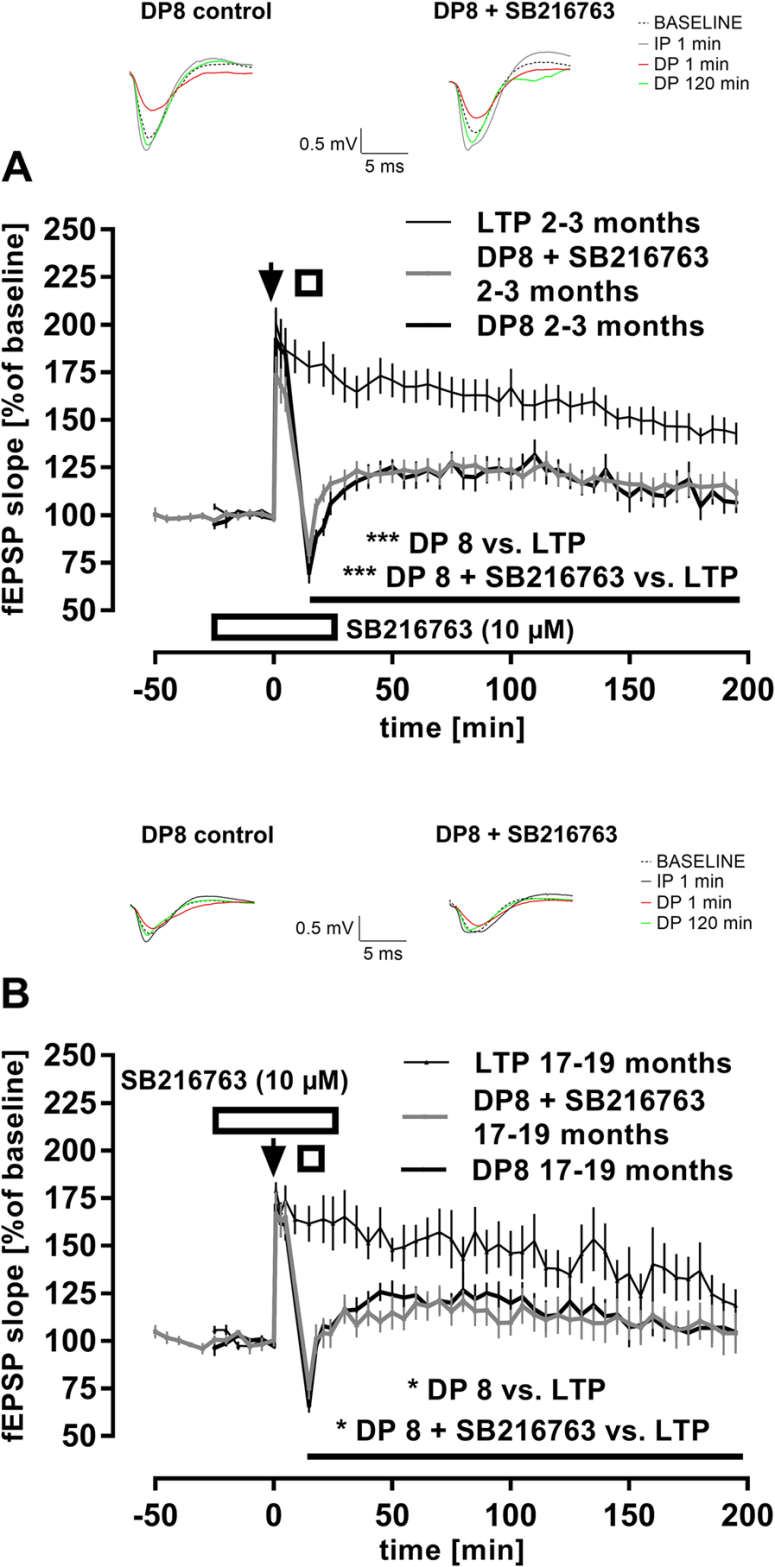
Hippocampal depotentiation does not rely on GSK3β. (**a**) The GSK3 inhibitor SB216763 (10 μM; represented by an open rectangle) was added 30 min prior to TBS application until 15 min after DP induction in a subset of hippocampal slices from 2 to 3 month-old- C57Bl/6 J mice (*n* = 11). No significant effect was observed compared to DP controls (*n* = 9) (*p* = 0.614). DP was clearly induced in both conditions since highly significant differences were detected when compared to LTP controls (n = 10) (DP 8: *F*_(1, 17)_ = 19.71, ***p = 0.0004; DP 8 + SB216763: *F*_(1, 19)_ = 21.49, ***p = 0.0002). Arrow represents single TBS, open square represents TPS. Error bars represent SEM. Traces show representative examples of field excitatory postsynaptic potentials (fEPSP) recorded during baseline, 1 min post-TBS delivery (IP 1 min), 1 min post-TPS delivery (DP 1 min), and 120 min post-TPS delivery (DP 120 min). Calibration bars: 0.5 mV and 5 ms. (**b**) Replicating same experiments in aged (17–19 month-old) C57Bl/6 J mice (n = 7) equally failed to show significant effects of SB216763 (10 μM; represented by an open rectangle) on DP compared to DP controls (*n* = 6) (*p* = 0.857). When compared to LTP (n = 6), either DP condition showed a significant LTP reversal (DP 8: *F*_(1, 10)_ = 6.193, *p = 0.0321; DP 8 + SB216763: *F*_(1, 11)_ = 7.458, *p = 0.0195). Arrow represents single TBS, open square represents TPS. Error bars represent SEM. Traces show representative examples of field excitatory postsynaptic potentials (fEPSP) recorded during baseline, 1 min post-TBS delivery (IP 1 min), 1 min post-TPS delivery (DP 1 min), and 120 min post-TPS delivery (DP 120 min). Calibration bars: 0.5 mV and 5 ms

Electrically-induced NMDAR-LTD was demonstrated to be closely dependent on GSK3ß activation [2, 54, 55]. Since DP shares several commonalities with LTD including induction mechanisms such as the reliance on NMDAR activation (reviewed in [61]), we tested whether our DP8 protocol induces a DP that also requires GSK3ß activation. Thus, we conducted a series of experiments in which the GSK3 inhibitor SB216763 was applied to slices of wild-type mice. Because of age-dependent effects of GSK3β on synaptic plasticity [55], we evaluated the effects of SB216763 in adult (2–3 months) and aged (17–19 months) mice. As shown in Fig. 2a, SB216763 (10 μM, *n* = 11) failed to show any effects on DP of adult mice when compared to DP8 controls (*n* = 9) (main effect of *compound*_**WT + SB216763** × WT_: *p* = 0.614; main effect of *time*: F _(38, 684)_ = 13.57, *p* < 0.001; no interaction *compound* × *time*). Consistently, both ‘DP 8’ and ‘DP 8 + SB216763’ induced robust LTP reversal when compared to LTP controls (post-TBS delivery; ‘DP 8 + SB216763’- main effect of *conditioning*_**DP 8 + SB216763** × LTP_: F _(1, 19)_ = 21.49, *p* = 0.0002; main effect of *time*: F _(41, 779)_ = 21.13, *p* < 0.0001; interaction *conditioning* × *time*: F _(41, 779)_ = 71. 119, *p* < 0.0001; ‘DP 8 only’- main effect of *conditioning*_**DP 8** × LTP_: F _(1, 17)_ = 19.71, *p* = 0.0004; main effect of *time*: F _(41, 697)_ = 23.42, p < 0.0001; interaction *conditioning* × *time*: F _(41, 697)_ = 9.423, *p* < 0.0001).

**Fig. 3:**
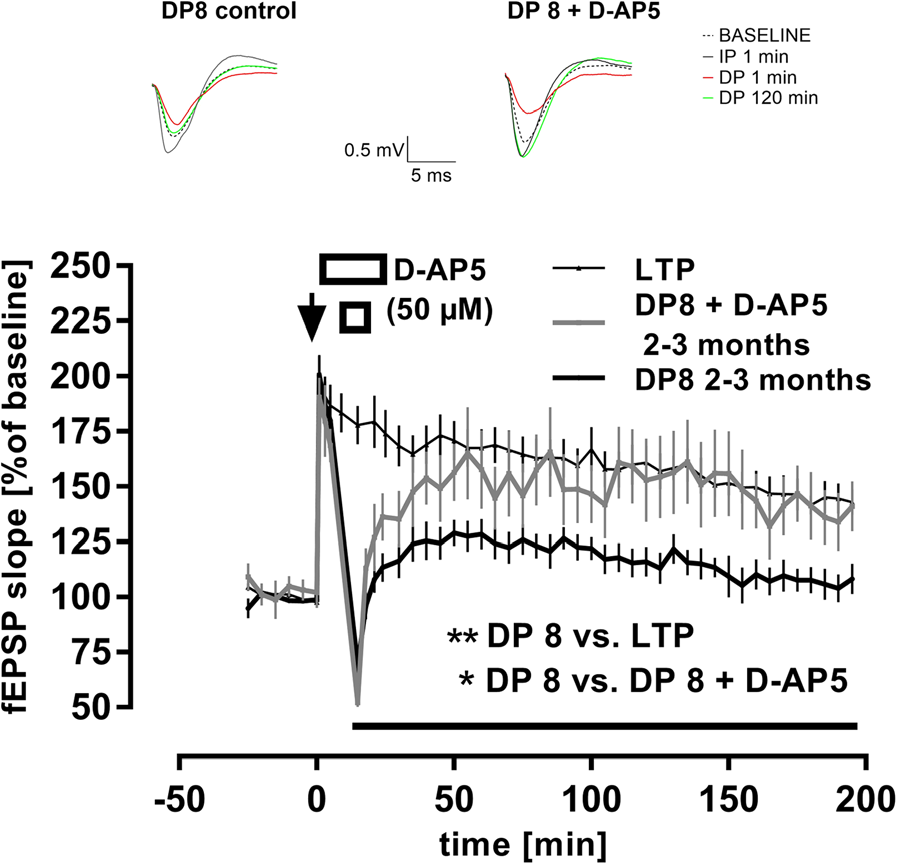
Hippocampal depotentiation depends on NMDAR activation. Application of the NMDAR antagonist D-AP5 (50 μM, n = 6) before DP induction resulted in residual fEPSP potentiation being significantly higher than in DP controls (n = 7) (F _(1, 11)_ = 4.945, *p = 0.048). Compared to LTP controls (n = 10), the main conditioning effect of DP 8 was clearly signficant (F _(1, 15)_ = 16.5, **p = 0.001), while this was not the case for slices bathed in D-AP5 (*p* = 0.3104). Error bars represent SEM. Traces show representative examples of field excitatory postsynaptic potentials (fEPSP) recorded during baseline, 1 min post-TBS delivery (IP 1 min), 1 min post-TPS delivery (DP 1 min), and 120 min post-TPS delivery (DP 120 min). Calibration bars: 0.5 mV and 5 ms

The same was true for the identical application of SB216763 in aged mice (*n* = 7) compared to age-matched DP8 controls (*n* = 6) (Fig. 2b; main effect of *compound*_**WT + SB216763** × WT_: *p* = 0.857; main effect of *time*: F _(46, 506)_ = 2.888, *p* < 0.001; no interaction *compound* × *time*). Of note, the application of SB216763 in adult mice seemingly reduced the magnitude of initial potentiation after TBS (IP). However, this difference was not statistically significant when compared to IP of DP controls (5 min recordings post-TBS; main effect of *compound*_**WT + SB216763** × WT_: *p* = 0.263; main effect of *time*: F _(2, 36)_ = 55.85, *p* = 0.007; no interaction *compound* × *time*). Furthermore, similarly to adult mice, also here both ‘DP 8’ and ‘DP 8 + SB216763’ induced robust LTP reversal when compared to aged-matched LTP controls (post-TBS delivery; ‘DP 8 + SB216763’- main effect of *conditioning*_**DP 8 + SB216763** × LTP_: F _(1, 11)_ = 7.458, *p* = 0.0195; main effect of *time*: F _(41, 451)_ = 7.95, *p* < 0.0001; interaction *conditioning* × *time*: F _(41, 451)_ = 2.935, p < 0.0001; ‘DP 8 only’- main effect of *conditioning*_**DP 8** × LTP_: F _(1, 10)_ = 6.193, *p* = 0.0321; main effect of *time*: F _(41, 410)_ = 8.25, p < 0.0001; interaction *conditioning* × *time*: F _(41, 410)_ = 2.764, *p* < 0.0001). Since the same batch and dose of SB216763 effectively inhibited LTD in middle-aged C57Bl/6 J mice (data not shown), we concluded that GSK3ß is not involved in DP. In contrast, we confirmed that TPS-induced DP depends on NMDAR activation (Fig. 3) [16, 34, 53]. Although application of the competitive NMDAR antagonist D-AP5 (50 μM) did not interfere with the transient depression of fEPSPs that followed DP8 delivery (mean fEPSP potentiation *1 min* after ‘DP8 only’ = 69 ± 7%, *n* = 7; mean fEPSP potentiation *1 min* after ‘DP8 + D-AP5’ = 52 ± 7%, *n* = 6; *p* = 0.119, not depicted), it blocked DP as evaluated by RM-ANOVA of whole recordings post- TPS delivery (main effect of *drug*_**DP 8 + D-AP5** × DP 8_: F _(1, 11)_ = 4.945, *p* = 0.048; main effect of *time*: F _(38, 418)_ = 10.64, *p* < 0.0001; interaction *drug* × *time*: F _(38, 418)_ = 1.696, *p* = 0.007). Consistently, fEPSP residual potentiation from the ‘DP8 only’ group of slices was, as expected, significantly lower when compared to LTP controls (*n* = 10) (post-TBS delivery; main effect of *conditioning*_**DP 8** × LTP_: F _(1, 15)_ = 16.5, *p* = 0.001; main effect of *time*: F _(41, 615)_ = 39.4, *p* < 0.0001; interaction *conditioning* × *time*: F _(41, 615)_ = 11.81, p < 0.0001). However, this was not the case for slices bathed in D-AP5 (post-TBS delivery; main effect of *conditioning*_**DP 8 + D-AP5** × LTP_: *p* = 0.3104; main effect of *time*: F _(41, 574)_ = 10.3, p < 0.0001; interaction *conditioning* × *time*: F _(41, 574)_ = 8.54, *p* < 0.0001).

### DP is unaltered in 14-month-old APPPS1–21 transgenic mice

**Fig. 4:**
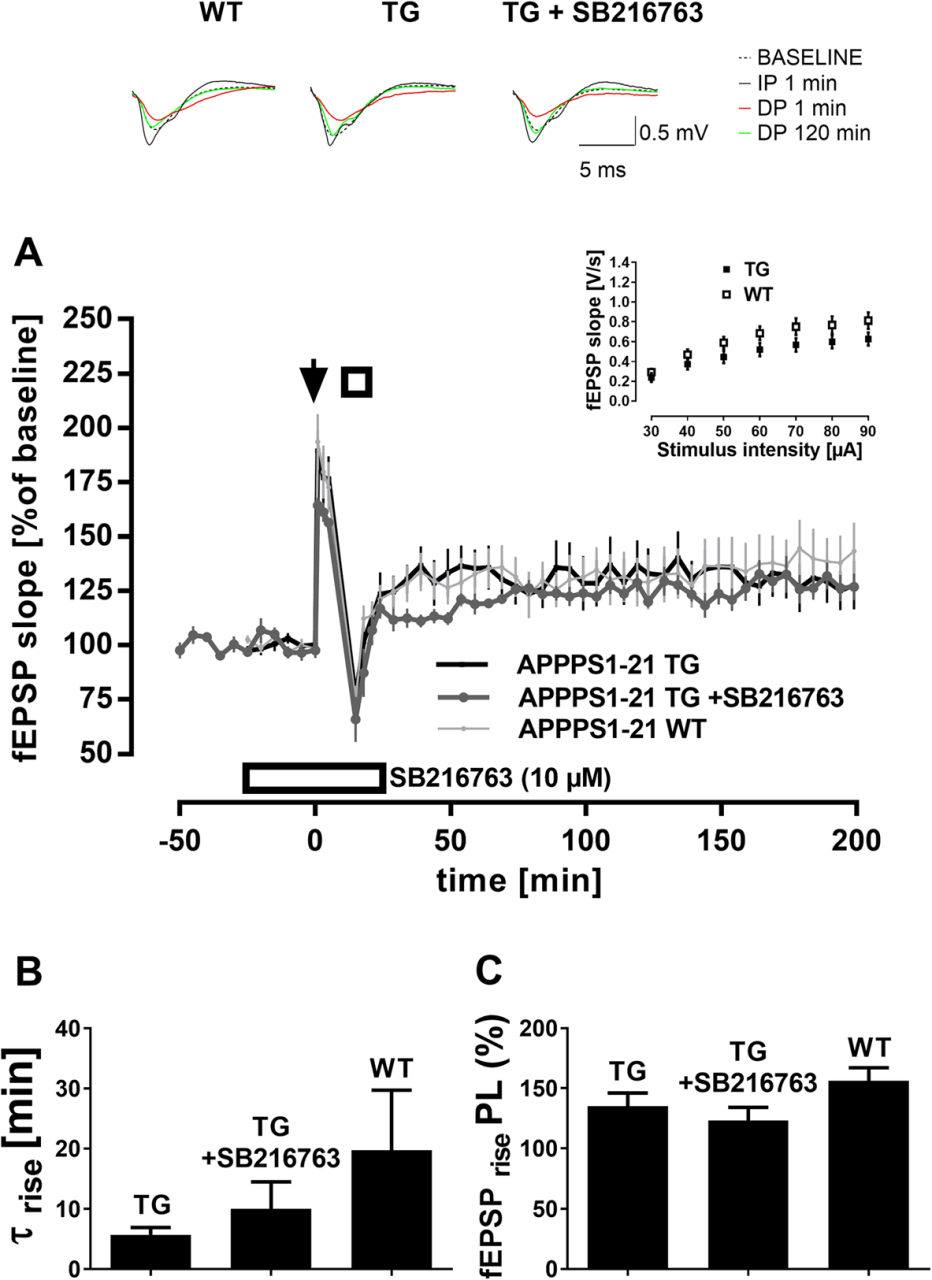
Hippocampal depotentiation is unaltered in APPPS1–21 transgenic mice. (**a**) DP was normal in 14-month-old APPPS1–21 transgenic mice (TG, n = 9) compared to APPPS1–21 wild-type littermates (WT, n = 9) (*p* = 0.901). Likewise, there were also no significant differences in basal synaptic transmission (inset; *p* = 0.165) or initial fEPSP potentiation after TBS (*p* = 0.946). Bath-application of the GSK3 inhibitor SB216763 (10 μM, *n* = 5) in a subset of APPPS1–21 TG slices did not alter their DP profiles (*p* = 0.507). Arrow represents single TBS, open square represents TPS, open rectangle represents drug application. Error bars represent SEM. Traces show representative examples of field excitatory postsynaptic potentials (fEPSP) recorded during baseline, 1 min post-TBS delivery (IP 1 min), 1 min post-TPS delivery (DP 1 min), and 120 min post-TPS delivery (DP 120 min). Calibration bars: 0.5 mV and 5 ms. Curve-fitting of DP data confirms the results of RM-ANOVAs. Mean values for τ _rise_ (**b**) were statistically similar between slices from APPPS1–21 TG, APPPS1–21 TG + SB216763 and their wild-type littermates (APPPS1–21 WT). Likewise, mean ‘plateau levels’ (**c**) were statistically indistinguishable between genotypes or conditioning groups

To characterize basic synaptic transmission in APPPS1–21 transgenic mice (TG), we first established input/output curves (30 to 90 μA). Previously, reports on the integrity of this parameter in the APPPS1–21 model of amyloidosis were contradictory, with evidence of deficient synaptic transmission seen in autaptic neurons [56], but not in aged mice *in vivo* [17]. In our settings, fEPSPs slopes from both genotypes increased proportionally to stimulus intensity, with I/O profiles of APPPS1–21 TG and APPPS1–21 WT not being significantly different (inset Fig. 4a; main effect of *genotype*: *p* = 0.165; main effect of *stimulus intensity*: F _(6, 114)_ = 48.7, *p* < 0.0001; no interaction *genotype* × *stimulus intensity*).

Next, we evaluated DP (Fig. 4a). Firstly, it must be noted that IP after TBS application was similar in slices from APPPS1–21 TG (*n* = 9) and APPPS1–21 WT (n = 9) (5 min recordings post-TBS; main effect of *genotype*: *p* = 0.946; main effect of *time*: F _(2, 32)_ = 8.013, *p* = 0.001; no interaction *genotype* × *time*). After DP8 delivery, APPPS1–21 TG displayed same DP when compared to APPPS1–21WT, with both genotypes showing similar levels of residual potentiation of fEPSPs along the whole duration of experiments (main effect of *genotype*: *p* = 0.901; main effect of *time*: F _(38, 608)_ = 8.551, *p* < 0.0001; no interaction *genotype* × *time*). To examine whether the progressing AD pathology might have brought GSK3ß into play [7, 39], in this way covertly altering DP profiles of APPPS1–21 TG, we applied the GSK3 inhibitor SB216763 (10 μM) to a subset of transgenic mice slices (*n* = 5). Initial potentiation after TBS seemed diminished in slices from treated APPPS1–21 TG compared to APPPS1–21 WT, but this difference was not significant (5 min recordings post-TBS; main effect of *genotype*: *p* = 0.256; main effect of *time*: F _(2, 24)_ = 6.303, *p* = 0.006; no interaction *genotype* × *time*). DP in treated and untreated APPPS1–21 TG were statistically indistinguishable (main effect of *compound*_**APPPS1–21 TG + SB216763** × APPPS1–21 TG_: *p* = 0.564; main effect of *time*: F _(38, 456)_ = 9.303, *p* < 0.0001; no interaction *compound* × *time*). Likewise, DP in treated APPPS1–21 TG slices was not significantly different from APPPS1–21 WT (main effect of *genotype*_**APPPS1–21 TG + SB216763** × APPPS1–21 WT_: *p* = 0.507; main effect of *time*: F _(38, 456)_ = 6.848, p < 0.0001; no interaction *genotype* × *time*).

Curve-fitting of DP data confirmed the above results, as no significant differences emerged through this analysis (Table 1). Mean values for τ _rise_ were not significantly different between groups of slices, reflecting comparable rise time-constants after DP8-induced depression of fEPSPs (*genotype* effect _**APPPS1–21 TG** × APPPS1–21 WT_: *p* = 0.220; *genotype* effect _**APPPS1–21TG + SB216763** × APPPS1–21 WT:_ *p* = 0.412, Fig. 4b). Mean ‘plateau levels’ at which fEPSPs stabilized were also comparable between genotypes (*genotype* effect _**APPPS1–21 TG** × APPPS1–21 WT_: *p* = 0.216; *genotype* effect _**APPPS1–21 TG + SB216763** × APPPS1–21 WT:_ *p* = 0.070, Fig. 4c).

**Table 1:** Curve-fitting of DP data of Alzheimer’s disease mouse models

Mice	Mean τ _rise_ (min)	Mean ‘plateau level’ (% of baseline)
APPPS1–21 WT	19.7 ± 10	156 ± 11
APPPS1–21 TG	5.7 ± 1	135 ± 11
APPPS1–21 TG +SB216763	10 ± 4.5	124 ± 11
THY-tau 22 WT	21.3 ± 13	141 ± 7
THY-tau 22 TG	6.3 ± 1	118 ± 5
THY-tau 22 TG +SB216763	3.8 ± 1	116 ± 4

### DP is enhanced in 12-month-old THY-Tau22 transgenic mice

**Fig. 5:**
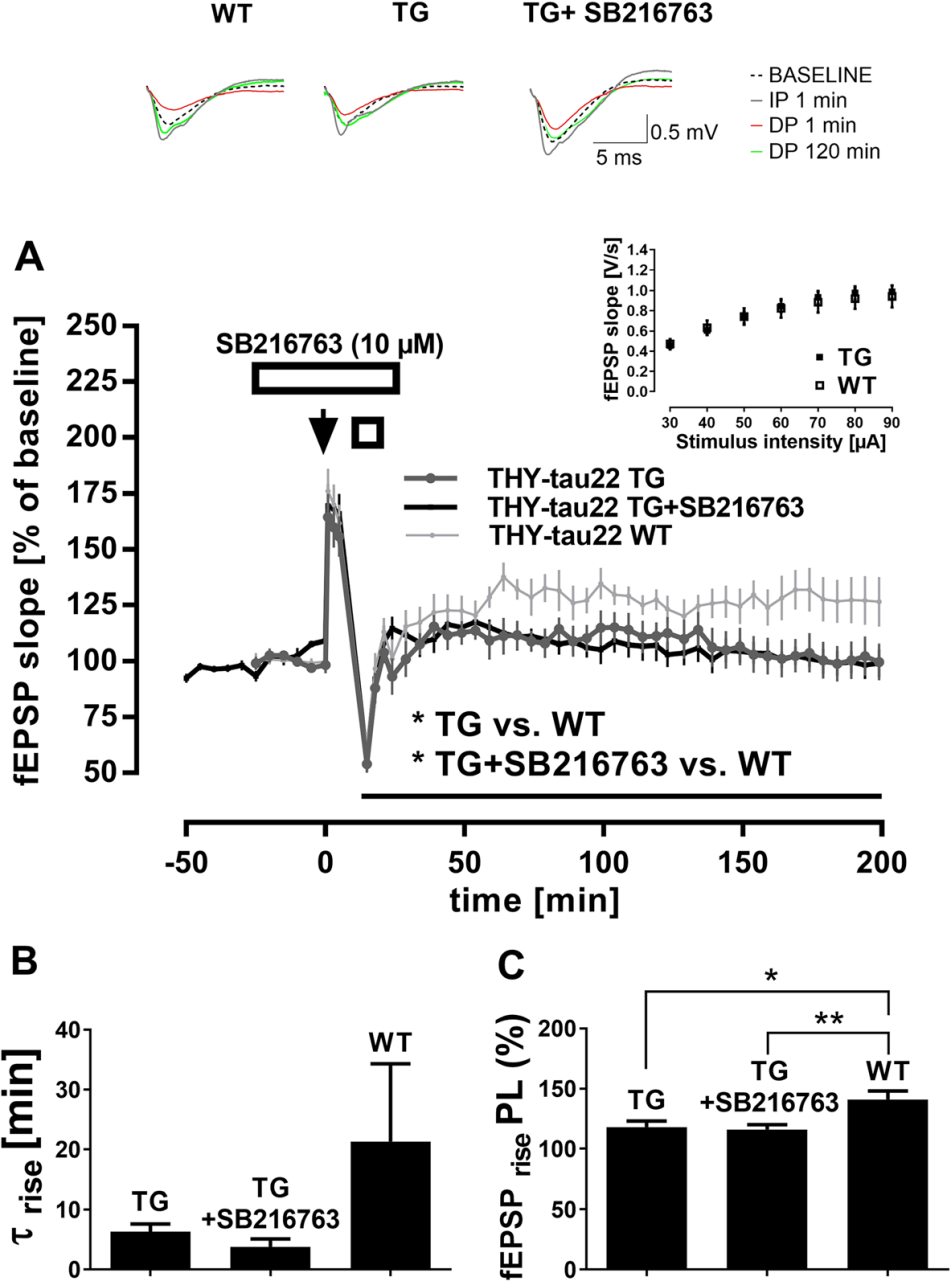
Hippocampal depotentiation is enhanced in THY-tau22 transgenic mice. (**a**) DP was significantly stronger in 12-month-old THY-tau22 transgenic mice (TG, *n* = 8) compared to THY-tau22 wild-type littermates (WT, n = 8) (F _(1, 14)_ = 5.235, *p = 0.038), while no significant differences were observed in basal synaptic transmission (inset; *p* = 0.859) or the initial fEPSP potentiation after TBS (*p* = 0.482). This abnormal enhancement of DP in THY-tau22 TG could not be rescued by bath-application of the GSK3β inhibitor SB216763 (10 μM, n = 6), since a significant difference between treated slices and WT controls was still evident (F _(1, 12)_ = 8.21, *p = 0.014). Arrow represents single TBS, open square TPS, and open rectangle drug application. Error bars indicate SEM. Traces show representative examples of field excitatory postsynaptic potentials (fEPSP) recorded during baseline, 1 min post-TBS delivery (IP 1 min), 1 min post-TPS delivery (DP 1 min), and 120 min post-TPS delivery (DP 120 min). Calibration bars: 0.5 mV and 5 ms. Curve-fitting of DP data extended the results of RM-ANOVAs. Mean values for τ _rise_ (**b**) were statistically similar between slices from transgenic mice (TG or TG + SB216763) and their wild-type littermates (WT). In contrast, mean ‘plateau levels’ (**c**) were significantly lower in THY-tau22 TG or THY-tau22 TG + SB216763 compared to THY-tau22 WT (TG × WT: t (12.6) = 2.752, *p = 0.017; TG + SB216763 × WT: t (10.9) = 3.113, **p = 0.010)

First, we analyzed input-output properties of THY-Tau22 transgenic mice (TG; *n* = 15) at the age of 12 months. At this age, these mice present severely deficient hippocampus-dependent learning and signs of synaptic dysfunction such as impaired LTD [2, 35, 73]. However, we did not detect discernible differences in basal synaptic transmission between THY-Tau22 TG and THY-Tau22 WT (*n* = 10) (Fig. 5a, inset; main effect of *genotype*: *p* = 0.859; main effect of *stimulus intensity*: F _(6, 138)_ = 89.58, *p* < 0.0001; no interaction *genotype* × *stimulus intensity*). When synaptic plasticity was examined (Fig. 5a), IP following TBS delivery did not significantly differ between genotypes (THY-Tau22 TG, *n* = 8; THY-Tau22 WT, n = 8) (5 min recordings post-TBS; main effect of *genotype*: *p* = 0.482; main effect of *time*: F _(2, 28)_ = 10.12, *p* = 0.0005; no interaction *genotype* × *time*). However, there was a significant difference between genotypes after DP induction. In THY-Tau22 TG there was a strong reduction in the residual fEPSP potentiation that is typical of DP with the DP8 protocol (see results 3.1–3.3 above). In contrast, this residual potentiation was promptly observed in THY-Tau22 WT (main effect of *genotype*_**Tau22 TG** × Tau22 WT_: F _(1, 14)_ = 5.235, *p* = 0.038; main effect of *time*: F _(38, 532)_ = 9.638, *p* < 0.0001; no interaction *genotype* × *time*).

In a previous investigation [2], we found that a deficit in LTD in 10–12 month-old THY-Tau22 TG could be rescued by bath-application of the GSK3β inhibitor SB216763 (10 μM). Therefore, we examined whether the same compound could normalize the altered DP in these mice as well (THY-Tau22 TG + SB216763, *n* = 6). However, DP in treated slices of THY-Tau22 TG was indistinguishable from DP in untreated THY-Tau22 TG (main effect of *compound*_**Tau22 TG + SB216763** × Tau22 TG_: *p* = 0.948; main effect of *time*: F _(38, 456)_ = 12.81, p < 0.0001; no interaction *compound* × *time*), and still significantly enhanced compared to DP of THY-Tau22 WT controls (main effect of *genotype*_**Tau22 TG + SB216763** × Tau22WT_: F _(1, 12)_ = 8.21, *p* = 0.014; main effect of *time*: F _(38, 456)_ = 7.142, p < 0.0001; interaction *genotype* × *time*: F _(38, 456)_ = 1.536, *p* = 0.024).

Curve-fitting of DP data confirmed the results of above RM-ANOVAs (see Table 1). Mean values for τ _rise_ were not significantly different between groups of slices, reflecting comparable rise time-constants after DP8-induced depression of fEPSPs (*genotype* effect _**Tau22 TG** × Tau22 WT_: *p* = 0.288; *genotype* effect _**Tau22 TG + SB216763** × Tau22 WT:_ *p* = 0.222, Fig. 5b). However, mean ‘plateau levels’ at which fEPSPs stabilized were significantly lower in THY-Tau22 TG or THY-Tau22 TG + SB216763 when compared to THY-Tau22 WT (*genotype* effect _**Tau22 TG** × Tau22 WT_: t (12.6) = 2.752, *p* = 0.017; *genotype* effect _**Tau22 TG + SB216763** × Tau22 WT:_ t (10.9) = 3.113, *p* = 0.010, Fig. 5c). To note, when curve-fitting results of the APPPS1–21 and THY-tau 22 groups are evaluated together, there was a strikingly high variability in τ _rise_ of WT animals, which was not observed in Tg animals, the reason of which is unclear.

## Discussion

### TPS generates stable DP in mice with increasing inducibility with age

DP has long been described [18], but a procedure for the induction of DP in mouse slices employing stimuli that mimic the hippocampal theta rhythm (TBS and TPS; see [6]), and provide data about the initial time-course of LTP before DP induction, had not been established before. In our reference group (2–3 month-old mice), LTP reversal was consequent to the duration/amount of TPS, corroborating previous reports [53, 70]. The most significant DP levels were obtained with 8 min continuous TPS (DP8). Our parametric studies show also that DP is modulated in an age-dependent manner. While in 2–3-month-old mice, ‘saturated’ levels of DP are only reached with DP8, DP develops in nearly the same way in 6–9 month-old mice, regardless of the amount of TPS (from DP2 to DP8) employed. These findings extend previous studies in rat slices [22, 52].

### Activation of GSK3 is not involved in DP_NMDAR_ induction

The experiments with D-AP5 confirm that DP generated by our protocol requires activation of NMDAR, in agreement with previous investigations using other induction protocols [16, 25, 34, 48, 53]. On the other hand, inhibiting GSK3ß with SB216763 uncovers mechanistic differences between DP and LTD. Unlike the latter, which relies on the activation of GSK3ß [2, 55], DP does not depend on GSK3ß. Thus, apart from mutual induction mechanisms [24, 61], it seems obvious that downstream of NMDAR, the signaling mechanisms involved in the two types of synaptic plasticity bifurcate. In support of this supposition, it has been shown that LTD and DP differ regarding the identity of activated small GTPases and MAPKs involved in AMPARs endocytosis [80]. Importantly, in view of our results, the hypothesis about the role of GSK3ß in synaptic plasticity [54, 55] needs to be updated. One of its basic tenants, that GSK3ß functions as a ‘molecular switch’ between LTP and LTD, is based on experiments whereby GSK3ß activity was necessary for depression of synaptic transmission requiring NMDAR [55], and that upon GSK3ß inhibition (following LTP induction), NMDAR would be internalized [10]. However, we show here at least one form of activity-induced synaptic depression that does not require GSK3ß, occurs after LTP induction, and yet still depends on proper functioning of NMDAR, including metabotropic actions [34]. It is interesting in this context, that recent experiments from our laboratory indicate that inhibition of GSK3 may impair LTP, which is in contrast to previous studies [15, 20, 55].

### DP is unaffected in APPPS1–21 transgenic mice

Aged APPPS1–21 mice of comparable age as used in our study were shown earlier to present diffuse cerebral Aß deposition accompanied by neuritic abnormalities, neuroinflammation, but no significant neuronal loss in CA1 [60, 76]. Here we show that DP is intact in this AD mouse model of accelerated amyloidosis. Our findings seem at odds with two previous studies addressing the relationship between amyloidosis and DP. Previously, Kim et al. [31] demonstrated that the Aß-containing C-terminus of ß-APP (CT; the product of β-secretase cleavage of APP) reversed LTP when applied 10 min after LTP induction, while Huh et al. [26] showed that DP could not be induced in slices from aged Tg2576, a transgenic mouse model harboring the APP_695_SWE mutation. A very recent *in vivo* study by Qi et al. [57] reported that 4–6-month-old, anaesthetized, pre-plaque TG (McGill-R-Thy1-APP) rats developed only a transient DP at apical synapsed in the CA1 region upon LFS at 1 Hz, while the same stimulation caused complete LTP reversal in WT animals. Interestingly, DP could be rescued by application of an antibody that specifically binds Aß oligomers. However, critical methodological differences render a comparison difficult between the above and our results. For example, the acute action of exogenous Aβ peptides or fragments such as CT, used by Kim et al. [31] is considerably more deleterious to synaptic plasticity than the gradual exposure to endogenous Aβ that follows from the abnormal cleavage of APP in transgenic mice (see [66]). In Huh et al. [26], the resistance to DP was circumscribed only to aged Tg2576, and the contrast with our data could be the result of different transgenic mice employed as well as of the different paradigm to induce LTP (multiple episodes of 100 Hz stimulation). Noteworthy, the conserved DP of APPPS1–21 shown here contrasts sharply with an assessment of NMDAR-LTD done in parallel with our DP studies in the same batch of mice, under identical experimental conditions. In these experiments, we found LTD to be severely impaired (Ahmed et al. in preparation). The contrasting effects of Aβ-driven pathology on DP and LTD are especially revealing considering that DP and LTD are thought to regulate depression of synaptic transmission through partially overlapping mechanisms [24, 61]. Since pathologically elevated Aβ in related APP mouse models were previously shown to either abolish or enhance LTD (see ‘introduction’), we conjecture that such discrepancy could reflect a greater crosstalk between molecular effector mechanisms triggered by Aβ and those required preferentially for LTD. For example, at the level of NMDAR, ‘synaptotropic’ effects of Aβ appear to preferentially engage GluN2B-containing NMDAR [23, 28, 37, 59], which have been implicated in the induction of LTD [12, 49], but not of DP [38, 46]. Another example of a possible selective interaction of LTD mechanisms with Aβ is via GSK3β. Aβ overstimulates GSK3β activity [72], and reciprocally, GSK3β contributes to deleterious misprocessing of APP [39]. As noted, this protein kinase is necessary for NMDAR-dependent LTD [2, 55]. However, as shown here, GSK3β does neither seem to play a role in DP of WT mice, nor modulates DP profiles of APPPS1–21.

### DP magnitude is enhanced in the THY-Tau22 model of tauopathy

The primary finding of our study with THY-Tau22 mice is the significant enhancement of DP at the age of 12 months, which is the first demonstration of altered DP in a tauopathy mouse model. At this age, earlier studies demonstrated that these mice exhibit robust AD-like tauopathy including Tau hyperphosphorylation and pathological Tau phosphorylation, formation of intracellular neurofibrillary tangles-like Gallyas silver-positive inclusions, Tau filaments, ghost tangles, and gliosis, in several brain regions including CA1 [63]. Pathologically processed Tau endangers synaptic plasticity and cognition well before abnormal synaptic transmission due to synapse loss or neurodegeneration become prevalent [19, 21, 71, 79]. In transgenic mouse models such as THY-Tau22, mutations in (human) Tau foster abnormal phosphorylation at both physiological and pathological sites [29], which leads to both a loss of normal protein function and a gain in toxic effects [2, 21, 42, 44, 63]. While from the current data it is not possible to determine through which route DP would be abnormally enhanced, we demonstrate that tauopathy can produce specific changes in synaptic function leading, in turn, to enhanced DP. Our previous results with LTD [2, 73] and now with DP are particularly informative because no deficits in HFS-LTP were detected at this age in this mouse model [63], which could be suggestive of a greater susceptibility of activity-induced forms of synaptic depression to functional pathology due to tauopathy.

SB216763 did not normalize the increased magnitude of DP in slices from THY-Tau22 mice. The same compound, applied under identical experimental conditions as presented here, did rescue an impaired NMDAR-LTD [2]. This is indicative of a mechanistic difference in the pathology that underlies the changes in LTD and DP in THY-Tau22 mice. Importantly, this finding goes beyond the verification that GSK3ß is necessary for LTD but not for DP in WT mice because in THY-Tau22 mice SB216763 should have affected GSK3ß protein substrates and phosphorylation sites involved in Tau pathology [32], independently of a putative role of GSK3ß in the induction of DP. In fact, given the rescuing effect on LTD [2], we expected the same treatment could also normalize the DP phenotype in THY-Tau22.

## Conclusion

After refining our novel protocol for DP-induction, we found evidence that even apparently related types of synaptic plasticity such as LTD and DP are vulnerable to different parts of the complex pathological scenario that is progressing during AD and tauopathies. DP is specifically sensitive to pathological consequences of protein Tau hyperphosphorylation as modelled by mutant-Tau expressing THY-Tau22 mice [63], but not to accelerated amyloidosis present in APPPS1–21 mice [58]. Furthermore, in contrast to LTD [2], inhibition of GSK3ß does not impact DP induction in WT mice and does not rescue the DP phenotype of 12-month-old THY-Tau22 mice, a result that offers a cautionary tale on the use of GSK3ß inhibitors as therapeutic tools for the re-normalization of synaptic plasticity alterations in AD. The divergent degree of functional deficits obtained in LTD and DP in same AD transgenic mice advocates the use of DP together with LTD and LTP as a complementary tool for the detection of deficits in synaptic functions in order to further specify and characterize the synaptic phenotypes of different pathological mechanisms in AD.

## Data Availability

The datasets generated during this study are available from the corresponding author on reasonable request.
